# Supplementary material for: New Approach Methods for Hazard Identification: A Case Study with Azole Fungicides Affecting Molecular Targets Associated with the Adverse Outcome Pathway for Cholestasis
Source: Cells. 2022 Oct 19;11(20):3293. doi: 10.3390/cells11203293 (PMC9600068; doi:10.3390/cells11203293)
Supplement: Supplementary file 1 [file cells-11-03293-s001.zip › cells-1897235-supplementary.pdf]

Supplementary table S1: gene expression as measured by RT-PCR

Supplementary table 1: Changes in gene expression after treatment for 24h and 6 days with propiconazole (Pi) and tebuconazole (Te) in HepaRG cells. Fold changes, standard deviations and substance concentrations are given. Abbreviation: n.s., not significant.

	24 hours			6 days		
gene	Pi	Te	Pi + Te	Pi	Te	Pi + Te
hABCB11	0.47 ± 0.49 (40μM)	n.s.	0.08 ± 0.01 (40μM)	0.69 ± 0.08 (20μM)	n.s.	0.82 ± 0.08 (20μM)
hABCC3	0.82 ± 0.02 (40μM)	0.63 ± 0.28 (40μM)	0.84 ± 0.10 (40μM)	n.s.	n.s.	n.s.
hABCG5	0.18 ± 0.11 (40μM)	0.33 ± 0.23 (40μM)	0.10 ± 0.04 (40μM)	0.51 ± 0.26 (20μM)	n.s.	0.46 ± 0.05 (20μM)
hCYP7A1	0.09 ± 0.12 (40μM)	0.31 ± 0.01 (40μM)	0.23 ± 0.32 (20μM)	0.38 ± 0.02 (20μM)	0.88 ± 0.60 (20μM)	0.59 ± 0.16 (20μM)
hCYP8B1	0.11 ± 0.13 (40μM)	0.37 ± 0.21 (40μM)	0.28 ± 0.36 (20μM)	0.46 ± 0.12 (20μM)	n.s.	n.s.
hACAT2	1.96 ± 0.21 (20μM	2.23 ± 0.26 (20μM)	2.15 ± 0.32 (10μM)	2.05 ± 0.34 (20μM)	2.16 ± 0.77 (10μM)	2.04 ± 0.53 (10μM)
hHMGCR	1.68 ± 0.07 (20μM)	2.05 ± 0.25 (20μM)	1.94 ± 0.42 (10μM)	3.06 ± 0.89 (20μM)	2.04 ± 0.51 (10μM)	1.94 ± 0.16 (5μM)
hHMGCS1	2.04 ± 0.26 (20μM)	2.56 ± 0.63 (20μM)	2.32 ± 0.34 (10μM)	3.30 ± 0.25 (10μM)	2.88 ± 0.69 (10μM)	2.43 ± 0.32 (5μM)
hLSS	1.97 ± 0.09 (20μM)	2.33 ± 0.46 (20μM)	2.10 ± 0.40 (10μM)	3.15 ± 0.98 (20μM)	5.26 ± 1.26 (10μM)	5.88 ± 0.22 (20μM)
hOSTα	0.36 ± 0.44 (40μM)	0.56 ± 0.13 (40μM)	n.s.	0.28 ± 0.12 (20μM)	n.s.	0.42 ± 0.09 (20μM)
hOSTβ	n.s.	n.s.	n.s.	2.50 ± 0.92 (20μM)	n.s.	n.s.
hSC4mol	1.60 ± 0.17 (20μM)	1.81 ± 0.11 (20μM)	1.65 ± 0.23 (10μM)	3.03 ± 0.92 (20μM)	1.51 ± 0.29 (10μM)	1.55 ± 0.21 (20μM)
hSLC10A1	0.08 ± 0.10 (40μM)	0.34 ± 0.28 (40μM)	0.34 ± 0.47 (20μM)	0.37 ± 0.11 (20μM)	n.s.	n.s.
hSLCO1B1	0.63 ± 0.24 (40μM)	n.s.	n.s.	n.s.	n.s.	n.s.

Supplementary table S2: Proteindata

Supplementary table 2: Changes in protein amount after treatment for 24h with propiconazole (Pi) and tebuconazole (Te) in HepaRG cells. Fold changes and standard deviations are given.

	**protein amount**		
	Pi [40 μM]	Te [40 μM]	Pi + Te [40 μM]
ABCC3	0.48 ± 0.02	0.42 ± 0.01	0.47 ± 0.06
CYP8B1	0.35 ± 0.07	0.27 ± 0.01	0.29 ± 0.03
SLC10A1	0.50 ± 0.19	0.39 ± 0.17	0.51 ± 0.19

Supplementary table S3: DLR data based on Knebel et al. 2019

Supplementary table 3: Propiconazole and tebuconazole interact with different nuclear receptors in HepG2 cells. Here data on CAR and PXR are presented. Substance, concentrations, fold changes ≥ 2 or ≤ 0.75 and significance ($p < 0.05$, Mann-Whitney rank sum test, n.s.), and are given. Abbreviation: n.s., not significant.

Reporter	Substance	Concentration	Response	Significance
CAR	Pi	5µM	2,76	n.s.
	Pi	10µM	4,51	n.s.
	Pi	20µM	6,62	p < 0.05
	Pi	40µM	6,76	p < 0.05
	Te	5µM	0,97	n.s.
	Te	10µM	0,95	n.s.
	Te	20µM	0,73	n.s.
	Te	40µM	0,70	n.s.
PXR	Pi	5µM	2,79	n.s.
	Pi	10µM	5,31	p < 0.05
	Pi	20µM	8,77	p < 0.05
	Pi	40µM	9,33	p < 0.05
	Te	5µM	3,12	n.s.
	Te	10µM	5,40	p < 0.05
	Te	20µM	6,23	p < 0.05
	Te	40µM	7,00	p < 0.05

Supplementary table S4: Sequences of primers used for RT-PCR analysis

gene	forward primer (5'-3')	reverse primer (5'-3')
hABCB11	ACCGAGGTTGGAAAAGGTTGT	CAGCCAACGACCCTGTGAAT
hABCC3	CTGGTGGTTCACAAAGATGGC	GGCATTTTTCCCAGGTGCTG
hABCG5	GGGTGCTTGTTGGATCTGGA	CATTTGAGCTGCCACAAGTGA
hCYP7A1	CTTCTGCGAAGGCATTTGGG	GTCTGTCCCGCCTTGTAAGA
hCYP8B1	GGGAGGTTCTTTGCACTCAG	TAGTGGTGTGTCAGGGTC
hACAT2	TATGAGCAAGGCTCCTCACTTG	TCAAAATGGCCAGCTTTCTGTG
hHMGCR	GACGTGAACCTATGCTGGTCAG	GGTATCTGTTTCAGCCACTAAGG
hHMGCS1	AAGTCACACAAGATGCTACACCG	TCAGCGAAGACATCTGGTGCCA
hLSS	CGTGGTATATGCGCTCCTCA	AGATCTGTGAGCCGTTGGTG
hOSTα	TGGAACTTGCCCTCACTAGC	GGCAAAGGGTGTTCTTGTA
hOSTβ	TGCTGGAAGAGATGCTTTGGT	CTGCTTGCCTGGATGCTTCT
hSC4mol	GCAAGATGCTTTGGTTGTGC	AATGGTCACCCATGCCCAAA
hSLC10A1	TGCGCTATGTCATCAAGGGAG	GCCACATTGAGGATGGTGGA
hSLCO1B1	ACTGATTCTCGATGGGTTGG	TATTTGGAGTTTGGGGCA

Supplementary table S5: Used plasmids and positive controls for reporter gene assays.

	**plasmid**	**positive control**
promoter assay		
CYP7A1	pGL14- CYP7A1	5 µM PMA (Sigma-Aldrich)
transactivation assays		
CAR	pGAL4/DBD-CAR/LBD(+3aa)<br>pGAL4-(UAS)5-TK-Luc	10 µM CITCO (Enzo life sciences)
FXR	pGAL4-FXR-LBD<br>pGAL4-(UAS)5-TK-Luc	10 µM GW4064 (Sigma-Aldrich)
PXR	pGAL4-PXR-LBD<br>pGAL4-(UAS)5-TK-Luc	10 µM SR12813 (Sigma-Aldrich)<br>10 µM Rifampicin (Sigma-Aldrich)
